# Hydroxyethylstarch impairs renal function and induces interstitial proliferation, macrophage infiltration and tubular damage in an isolated renal perfusion model

**DOI:** 10.1186/cc7726

**Published:** 2009-02-25

**Authors:** Lars Hüter, Tim-Philipp Simon, Lenard Weinmann, Tobias Schuerholz, Konrad Reinhart, Gunter Wolf, Kerstin Ute Amann, Gernot Marx

**Affiliations:** 1 Department of Anaesthesiology and Intensive Care Medicine, University of Jena, Erlanger Allee, Jena, 07747, Germany; 2 Department of Surgical Intensive Care, University of Aachen, Pauwelsstrasse, Aachen, 52074, Germany; 3 Department of Internal Medicine III, University of Jena, Erlanger Allee, Jena, 07747, Germany; 4 Department of Pathology, University of Erlangen, Universitätsstr., Erlangen, 91054, Germany

## Abstract

**Introduction:**

The aim of the study was to evaluate some of the underlying pathomechanisms of hydroxyethylstarch (HES) induced adverse effects on renal function using 24 porcine kidneys in an isolated perfusion model over six hours.

**Methods:**

Infusion of either 10% HES 200/0.5, 6% HES 130/0.42 or Ringer's lactate (RL) was performed to achieve an haematocrit of 20% in eight kidneys from four animals per group. Physiological and pathophysiological parameters were determined (including N-acetyl-beta-aminoglucosidase as a marker for lysosomal tubular damage). Histological investigations and immunohistological stainings of the kidneys were performed.

**Results:**

Initially after haemodilution, HES 130/0.42 and HES 200/0.5 reduced urine output compared with RL (*P* < 0.01). After six hours, N-acetyl-beta-aminoglucosidase was significantly higher in HES 200/0.5 (81 ± 23 U/L) compared with HES 130/0.42 (38 ± 12 U/L) and RL (21 ± 13 U/L; *P* < 0.001). Osmotic nephrosis-like lesions (OL) of the tubuli were present in all groups showing a significantly lower number of OL in RL (1.1 ± 0.4; *P* = 0.002) compared with both HES groups (HES 200/0.5 = 2.1 ± 0.6; HES 130/0.42 = 2.0 ± 0.5). Macrophage infiltration was significantly higher in HES 200/0.5 compared with HES 130/0.42 (1.3 ± 1.0 vs. 0.2 ± 0.04; *P* = 0.044). There was a significant increase in interstitial cell proliferation in the HES 200/0.5 group vs. HES 130/0.42 (18.0 ± 6.9 vs. 6.5 ± 1.6; *P* = 0.006) with no significant difference in RL (13.5 ± 4.0).

**Conclusions:**

We observed impaired diuresis and sodium excretion by HES and identified renal interstitial proliferation, macrophage infiltration and tubular damage as potential pathological mechanisms of HES-induced adverse effects on renal function using an isolated porcine renal perfusion model. Furthermore, we demonstrated that 10% HES 200/0.5 had more of a pro-inflammatory effect compared with 6% HES 130/0.42 and caused more pronounced tubular damage than 6% HES 130/0.42 and RL. OL were present in all groups, but to a lesser degree after RL administration.

## Introduction

Sepsis and septic shock are associated with both a relative and an absolute intravascular volume deficit [1]. Thus, adequate volume replacement to restore and maintain circulating plasma volume appears to be fundamental to improve organ perfusion and nutritive microcirculatory flow. It has been shown that early goal-directed fluid resuscitation in patients with severe sepsis and septic shock is associated with improved outcome [2]. Recently updated international guidelines indicate that there is no solid evidence for preferring either colloids or crystalloids for fluid replacement in patients with sepsis or septic shock [3]. Recommended goals are to achieve and sustain a central venous pressure of at least 8 mmHg (≥12 mmHg if the patient is mechanically ventilated) and to administer fluid challenges, initially 1 L of crystalloids or up to 0.5 L of colloids in 30 minutes, for as long as key haemodynamic parameters, such as arterial blood pressure and heart rate, are improving in patients with signs of hypovolaemia. The question of which type of solution should be used as volume replacement remains controversial [4]. Hydroxyethylstarch (HES) solutions are one group of volume replacement solutions (VRS) that can be trialled in the haemodynamically unstable patient, and recent developments include the introduction of new formulations and newly available HES products.

Adverse effects of HES administration on renal function have spurred ongoing research into the pathological mechanisms. Schortgen and colleagues showed HES to be an independent risk factor for acute renal failure in severe sepsis [5]. The methodology of this study has been questioned, although it was randomised and controlled [6-8]. Recently, in a German multicentre randomised controlled trial (efficacy of volume substitution and insulin therapy in severe sepsis (VISEP) study) it has been shown that the use of 10% HES 200/0.5 compared with Ringer's lactate (RL) in patients with severe sepsis or septic shock is associated with an increased need for renal replacement therapy [9]. In this study the cumulative dosage of 10% HES200/0.5 was significantly correlated with the need for renal replacement therapy.

Of note, the underlying pathomechanisms of the HES-induced renal injury could not yet be identified. Indeed, a recent large prospective observational study in over 3000 critically ill patients showed that in those with ICU stays of more than 24 hours, sepsis, heart failure and haematological cancer were all significantly associated with the need for dialysis or haemofiltration therapy, but volume replacement with HES was not [10]. Comparing the result with the data of the VISEP study, one important difference is the total amount of administered HES. In the VISEP study patients received HES for up to 21 days with a median cumulative dose of 70.4 ml/kg (interquartile range: 33.4 to 144.2 ml/kg), whereas in the observational study the median total amount of HES per patient was lower at 1000 ml (interquartile range: 500 to 2250 ml corresponding to a cumulative dose of less than 15 ml/kg).

In the situation of kidney transplantation, osmotic nephrosis-like histological lesions (OL) have been noticed retrospectively in kidney transplant recipients when HES was used for fluid resuscitation of donors who were brainstem dead [11]. Using HES 200/0.62, a detrimental effect on initial graft function could be demonstrated [12]. Histologically, OL were detected in most of the specimens.

Given the lack of data, the aim of the study was to investigate whether interstitial proliferation, macrophage infiltration and/or tubular damage could be potential pathomechanisms of HES-induced adverse effects on renal function using two different HES solutions in comparison to a crystalloid solution (RL). We selected 10% HES 200/0.5 because its negative impact on renal function was shown in the VISEP study and 6% HES 130/0.42 is one of the currently used HES solutions.

## Materials and methods

Female German landrace pigs (n = 12, mean weight = 44.0 ± 4.6 kg) were used and the principles of laboratory animal care were followed, based on the guidelines of the local Animal Care office. The study was approved by the local Animal Protection Committee and by the governmental Animal Care Office (Thüringer Landesamt für Lebensmittelsicherheit und Verbraucherschutz, Bad Langensalza, Germany). Animals were fasted for 24 hours with water *ad libitum *before the experiment.

### Infusion solutions

Three different VRS were studied: one crystalloid and two different HES solutions. RL as a crystalloid solution (Sterofundin, BBraun Melsungen, Germany) was compared with 10% HES 200/0.5 in 0.9% sodium chloride (NaCl; Hemohes 10%, BBraun, Melsungen, Germany) and 6% HES 130/0.42 in 0.9% NaCl (Venofundin 6%, BBraun, Melsungen, Germany).

### Kidney retrieval

Pigs were premedicated with ketamine (500 mg intramuscularly) to allow placement of an intravenous catheter in an auricular vein and to initiate pulse oximetry and continuous echocardiography (ECG) monitoring. Anaesthesia was induced by intravenous injection of propofol (2 to 3 mg/kg) and sufentanil (3 μg/kg) until intubating conditions were achieved. Pigs were orally intubated and placed in the supine position. Anaesthesia was maintained with a continuous infusion of propofol (20 to 35 mg/kg/hour). Controlled mode ventilation was chosen to ventilate the animals with an inspiratory oxygen fraction of 1.0, an inspiratory/expiratory ratio of 1:2 and a respiratory rate of 16 breaths/minute. The tidal volume was adjusted to maintain a partial pressure of arterial carbon dioxide (PaCO_2_) of 35 to 40 mmHg. The body core temperature was kept above 37°C by using an infrared lamp and warmed solutions.

Using sterile technique, the right external jugular vein and right carotid artery were surgically exposed. For blood sampling, an 8.5 F introducer sheath was inserted into the right external jugular vein and an arterial catheter for haemodynamic monitoring was placed into the right carotid artery.

Accordingly to a predefined random list, animals were allocated for fluid therapy with one of the three VRS. Thereafter, haemodilution was performed in the animals using either 500 mL of 10% HES 200/0.5 or 6% HES 130/0.42 solutions or 1000 mL of RL. Thereafter, 1 L of blood was collected by autologous normovolaemic haemodilution into a sterile receptacle containing 15,000 IU heparin and 5 mg verapamil. In all animals, a midline laparotomy was performed using standardised sterile surgical techniques and both kidneys were surgically removed 15 minutes after infusion of VRS and flushed immediately with 250 ml saline at a temperature of 37°C and a hydrostatic pressure of 100 cm.

### Isolated organ perfusion

**Figure 1:**
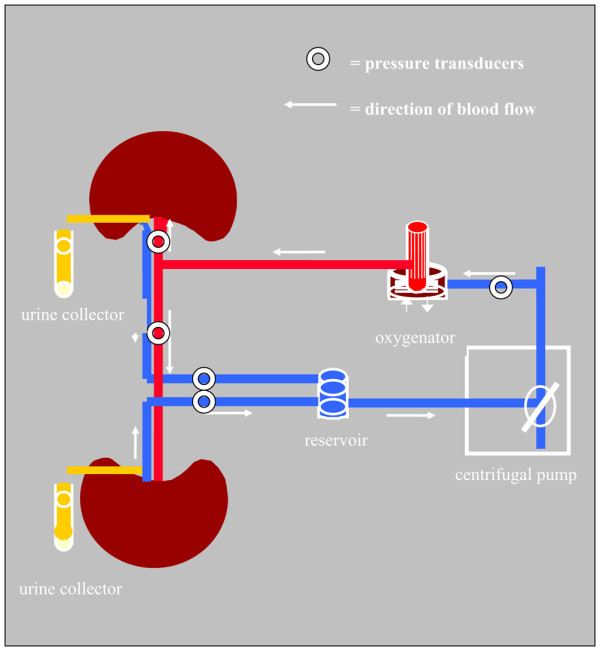
Schematic diagram of the isolated renal perfusion system.

Immediately after preparation the kidneys were reperfused on the isolated organ perfusion system (IOPS; Figure 1). The IOPS was designed using commercially available clinical-grade cardiopulmonary technology consisting of a centrifugal blood pump (0–40-00; Stöckert, Munich, Germany), a heat exchanger (ST II; Stöckert, Munich, Germany) and an oxygenator (Polystan Safe Micro; Maquet Cardiopulmonary, Hirrlingen, Germany). The gas flow was adjusted to achieve a partial pressure of arterial oxygen (PaO_2_) of 200 mmHg and a paCO_2 _of 30 mmHg. The circuit hardware included a TS 410 flow transducer (Transonic Systems Inc., Ithaka, NY, USA) and pressure transducers (BBraun, Melsungen, Germany). The circuit was primed with isotonic saline. To the circuit, 400 ml heparinised whole blood was added after priming and allowed to circulate at a temperature of 37°C. For the control of the haematocrit a continuous blood parameter monitoring system (CDI 500; Terumo Cardiovascular Systems, Ann Arbor, Mi, USA) was integrated in the IOPS.

### Experimental protocol

After stabilisation, the kidneys were perfused with one of the investigated VRS aimed at maintenance of a constant haematocrit of 20%. If 10% HES 200/0.5 was used for haemodilution during kidney retrieval *in vivo*, we continued to use 10% HES 200/0.5 for perfusion of the kidneys in the IOPS. The same applied for 6% HES 130/0.42 and RL. Renal blood flow, pressure and resistance were monitored continuously. Urine output was recorded hourly. From urine specimen, N-acetyl-beta-D-glucosamidase (beta-NAG; analysed by a spectrophotometric method; Hoffmann-La Roche, Basel, Switzerland), which is a sensitive marker of lysosomal tubular damage, was measured (normal range 0 to 7 U/L). Creatinine clearance was measured as an estimate of glomerular filtration rate (Cl_Crea _= U_crea _× U_vol_/P_crea _× duration of urine collection period; where U_crea _= urine creatinine concentration; U_vol _= urine volume during the collection period; P_crea _= serum creatinine concentration). Colloid osmotic pressure (COP) was analysed using a membrane colloid oncometer with a 20,000-Daltons semipermeable membrane (BMT 923; Delta-Pharma, Pfullingen, Germany). Blood gas analyses and urine sodium analyses were measured using a standard blood gas oximetry system (ABL 625; Radiometer, Copenhagen, Denmark) with a co-oximeter. All of the above named parameters were determined every two hours.

### Histological analysis

Needle-core biopsies were taken after perfusion, fixed in 4% formaldehyde and embedded in paraffin. Sections of 4 μm were cut and stained with H&E, Periodic Acid-Schiff and a fibrous tissue stain (Sirius red). Renal morphology was assessed semiquantitatively using the following four criteria of tubulointerstitial renal injury: acute tubular necrosis (dilation of tubuli with flattening or loss of the tubular epithelium) interstitial bleeding, interstitial inflammation and OL of the tubuli. KA, blinded to the VRS groups, scored each variable using a semiquantitative scoring system (0 to 4) for each criterion in 20 randomly sampled visual fields per animal: 0 (absent), 1 (0 to 25%), 2 (25 to 50%), 3 (50 to 75%) or 4 (more than 75%).

For further analysis immunohistochemisty was performed using antibodies against proliferating nuclear antigen (PCNA) for interstitial or glomerular cell proliferation and the marker for macrophage infiltration ED-1. KA, blinded to the VRS groups, assessed the number of positive cells per visual field in 20 randomly sampled visual fields per animal (positive cells/visual field (pc/vf)).

### Statistical analysis

Data were analysed using SPSS 13.0 for Windows (SPSS Inc., Chicago, IL, USA) and all results are presented as mean ± standard deviation. After verifying normal data distribution (skewness < 1.5) [13], effects of infusion solution and time were statistically analysed by analysis of variance (ANOVA) for repeated measurements with Bonferroni's correction for multiple comparisons (diuresis, sodium transport, Cl_Crea _and beta-NAG). All histological parameters were analysed using ANOVA with Bonferroni's correction. For the significance level of an alpha value of 0.05 an analysis of the prospective power of the experimental design was performed. A three-fold increase in beta-NAG between two groups was regarded as important. A sample size of at least seven kidneys in each group was necessary in order to achieve a power of 80%.

## Results

All animals and kidneys were analysed. The groups were comparable in regards to the mean arterial pressure and renal blood flow (Table 1). Overall, animals received 82.1 ± 24.5 ml/kg RL (22.9 ± 2.3 ml/kg during preparation and 59.2 ± 22.2 ml/kg during isolated renal perfusion), 20.0 ± 1.2 ml/kg HES 200/0.5 (11.9 ± 0.7 ml/kg during preparation and 8.1 ± 0.5 ml/kg during isolated renal perfusion) or 33.0 ± 7.6 ml/kg HES 130/0.42 (10.9 ± 1.2 ml/kg during preparation and 22.1 ± 5.4 ml/kg during isolated renal perfusion). This resulted in 2.0 ± 0.04 g/kg hydoxyethylstarch in group HES 200/0.5 and 2.0 ± 0.2 g/kg hydoxyethylstarch in group HES 130/0.42 during the whole experiment.

**Table 1:** Haemodynamic variables during isolated renal perfusion

	**Time (hours)**	**0**	**2**	**4**	**6**
**Blood flow** **(ml/g organ weight)**					
	**HES 200/0.5**	0.87 ± 0.15	0.92 ± 0.2	0.9 ± 0.24	0.98 ± 0.19
	**HES 130/0.42**	0.86 ± 0.12	0.87 ± 0.09	0.86 ± 0.08	0.96 ± 0.07
	**RL**	0.83 ± 0.18	0.94 ± 0.07	1.03 ± 0.19	0.98 ± 0.19
**MAP (mmHg)**					
	**HES 200/0.5**	69 ± 6	74 ± 6	79 ± 8	78 ± 10
	**HES 130/0.42**	72 ± 9	77 ± 12	76 ± 9	81 ± 7
	**RL**	77 ± 10^c^	69 ± 7^a^	73 ± 11	77 ± 9

Values are mean ± standard deviation. There were no statistical differences between groups.

HES = hydroxyethyl starch; MAP = mean arterial pressure; RL = Ringer's lactate.

### Functional parameters

**Figure 2:**
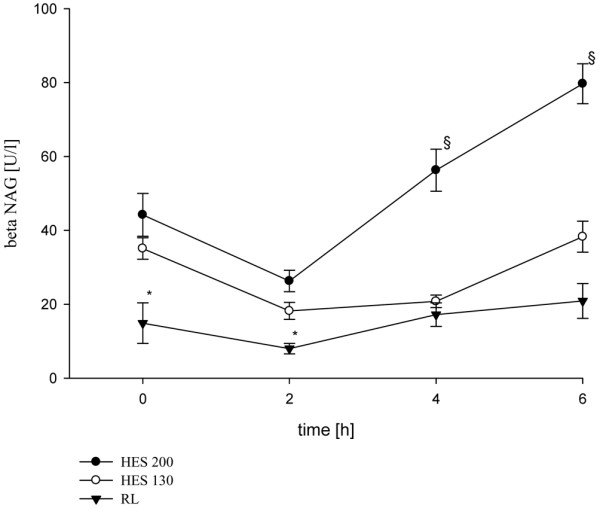
N-acetyl-beta-D-glucosamidase in urine as a marker of lysosomal tubular damage. Data are presented as mean ± standard deviation. Normal data are 0 to 7 U/L. * *P* < 0.05 RL *vs*. 6% hydroxyethyl starch (HES) 130/0.42 and 10% HES 200/0.5, § *P* < 0.001 10% HES 200/0.5 *vs*. Ringer's lactate (RL) and 6% HES 130/0.42. beta-NAG = N-acetyl-beta-D-glucosamidase.

**Figure 3:**
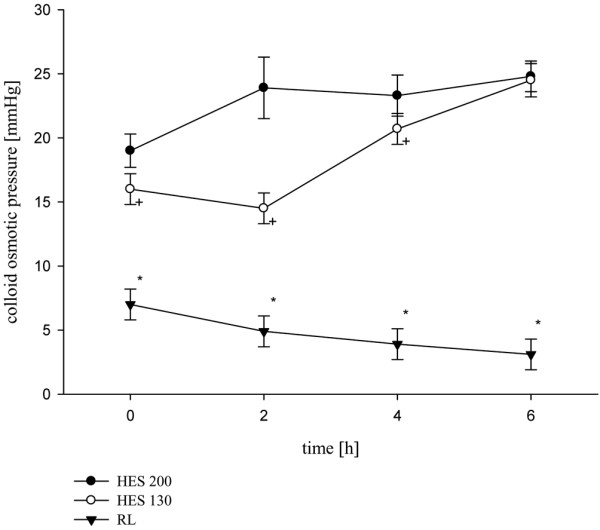
Values of colloid osmotic pressure over time between the three infusion groups. Data are mean ± standard deviation. *P < 0.001 Ringer's lactate (RL) *vs*. 6% hydroxyethyl starch (HES) 130/0.42 and 10% HES 200/0.5, + *P* < 0.05 6% HES 130/0.42 *vs*. 10% HES 200/0.5.

The mean diuresis during the study period was significantly higher over time in the RL group (16.2 ± 9.0 μl/minute/g) compared with HES 130/0.42 (3.6 ± 2.4 μl/min/g) and HES 200/0.5 (0.3 ± 0.5 μl/minute/g; *P* < 0.001; Table 2). Mean sodium transport over time was significantly lower in the HES 130/0.42 (-4.1 ± 0.8 mmol/minute/g) and HES 200/0.5 (-0.3 ± 0.2 mmol/minute/g) groups compared with RL (-13.9 ± 4.8 mmol/minute/g; *P* < 0.01; Table 2). During the study period mean Cl_crea _was significantly lower in the HES 200/0.5 group (0.01 ± 0.01 ml/minute/g) compared with the RL group (0.37 ± 0.06 ml/minute/g) and the HES 130/0.42 group (0.26 ± 0.05 ml/minute/g; *P* < 0.001; Table 2). After six hours beta-NAG as a marker of lysosomal tubular damage differed significantly in the HES 200/0.5 group (81.8 ± 23.4 U/L) compared with the HES 130/0.42 group (38.3 ± 11.8 U/L) and the RL group (20.9 ± 13.4 U/L; Figure 2). COP was different between groups; however, statistical significance between HES 200/0.5 and HES 130/0.42 disappeared after six hours (24.8 ± 3.4 mmHg vs. 24.5 ± 3.7 mmHg, respectively; Figure 3).

**Table 2:** Diuresis, creatinine clearance and sodium transport during isolated renal perfusion over the period of six hours

	**Time (hours)**	**0**	**2**	**4**	**6**
**Diuresis (μl/min/g)**					
	**HES 200/0.5**	0.6 ± 1.2	0.2 ± 0.3	0.2 ± 0.2^b^	0.2 ± 0.1^b^
	**HES 130/0.42**	3.0 ± 1.6	3.9 ± 2.6	3.6 ± 1.6	3.7 ± 1.6
	**RL**	30.4 ± 12.1^a^	20.2 ± 15.4^a^	5.0 ± 2.8	9.0 ± 5.6
**Cl_Crea _(ml/min/g)**					
	**HES 200/0.5**	0.01 ± 0.01	0.01 ± 0.01^d^	0.01 ± 0.01^d^	0.01 ± 0.01^d^
	**HES 130/0.42**	0.5 ± 0.07	0.33 ± 0.08	0.13 ± 0.02	0.09 ± 0.01
	**RL**	1.02 ± 0.12^c^	0.21 ± 0.03	0.12 ± 0.03	0.11 ± 0.05
**Tr_Na _(mmol/min/g)**					
	**HES 200/0.5**	-0.6 ± 0.5	-0.2 ± 0.1	-0.1 ± 0.07^b^	-0.1 ± 0.04^b^
	**HES 130/0.42**	-3.7 ± 0.9	-3.8 ± 0.8	-4.3 ± 0.7	-4.6 ± 0.7
	**RL**	-29.3 ± 5.8^c^	-15.4 ± 6.0^d^	-3.8 ± 1.0	-7.0 ± 6.5

Data are presented as mean ± standard deviation.

^a ^RL *vs*. 10% HES 200/0.5 and RL *vs*. 6% HES 130/0.42; *P* < 0.01

^b ^10% HES 200/0.5 *vs*. RL and 10% HES 200/0.5 *vs*. 6% HES 130/0.42; *P* < 0.01

^c ^RL *vs*. 6% HES 130/0.42 and RL *vs*. 10% HES 200/0.5; *P* < 0.001

^d ^10% HES 200/0.5 *vs*. RL and 10% HES 200/0.5 *vs*. 6% HES 130/0.42; *P* < 0.05

Cl_Crea _= creatinine clearance; HES = hydroxyethyl starch; RL = Ringer's lactate; Tr_Na _= sodium transport.

### Histological parameters

**Figure 4:**
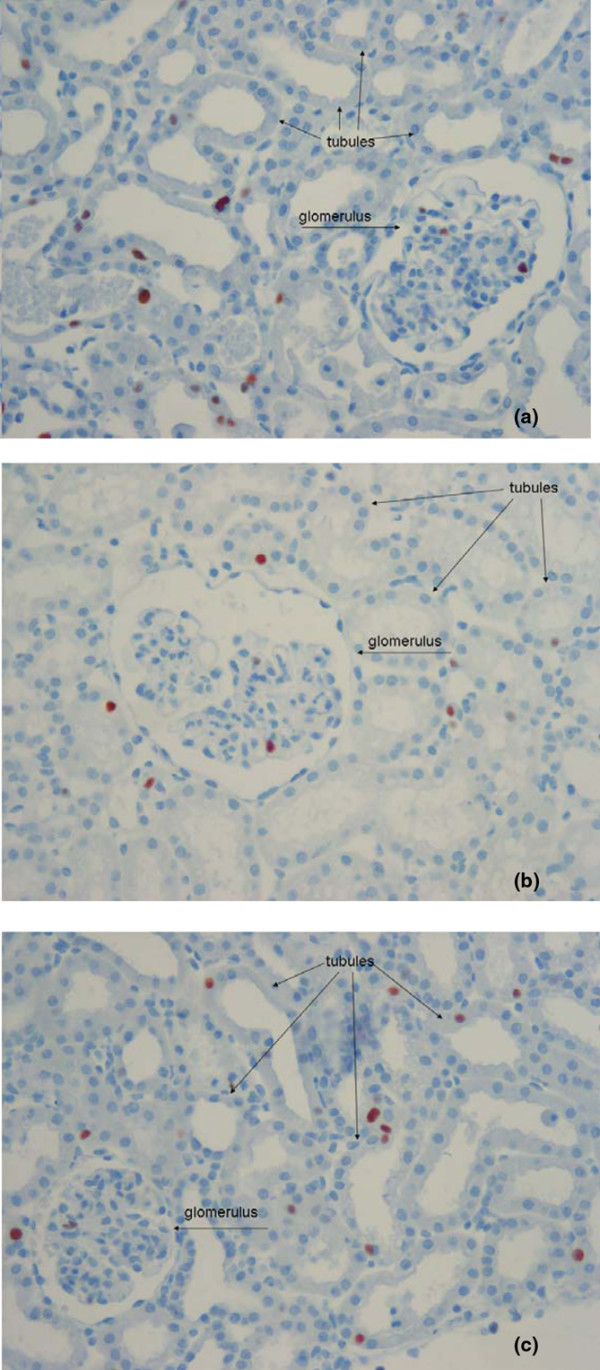
Representative histology sections of proliferating nuclear antigen stained kidneys. There was statistical less interstitial and glomerular cell proliferation in **(b)** 6% hydroxyethyl starch (HES) 130/0.42 compared with **(a)** 10% HES 200/0.5 and **(c)** Ringer's lactate (RL). Original magnification: × 200. PCNA = proliferating nuclear antigen.

**Figure 5:**
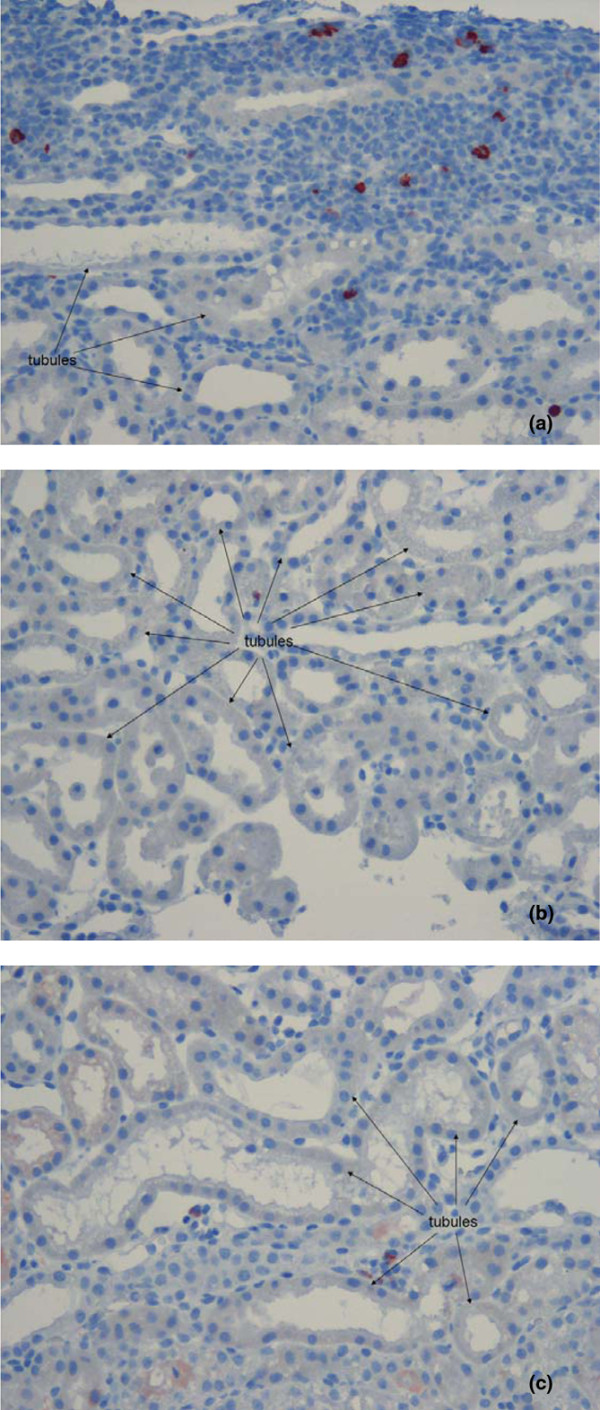
Representative findings of immunohistological staining of ED-1 positive interstitial macrophages. There was a significant higher number of infiltrating macrophages and also of other inflammatory cells in **(a)** 10% hydroxyethyl starch (HES) 200/0.5 compared with **(b)** 6% HES 130/0.42 and **(c)** Ringer's lactate (RL). Original magnification: × 200. ED-1 = marker for macrophage infiltration.

Histological examinations indicated that OL was present in all groups; there was, however, a significant difference with RL (1.1 ± 0.4) compared with HES 130/0.42 (2.0 ± 0.5) and HES 200/0.5 (2.1 ± 0.6; *P* < 0.01; Table 3). Cell proliferation (PCNA) in the HES 200/0.5 group (18.8 ± 7.1 pc/vf) was significantly higher than in HES 130/0.42 (7.2 ± 1.7 pc/vf; *P* = 0.008). There was no significant difference between PCNA and RL (14.1 ± 4.1 pc/vf). Comparison of interstitial and glomerular proliferation revealed significantly more pronounced interstitial cell proliferation: HES 200/0.5 (18.0 ± 6.9 pc/vf) vs. HES 130/0.42 (6.5 ± 1.6 pc/vf, *P* = 0.006). There was no significant difference with RL (13.5 ± 4.0 pc/vf; Table 3, Figure 4). The number of interstitial macrophages (ED-1) was significantly lower in the HES 130/0.42 group (0.2 ± 0.04 pc/vf) compared with the HES 200/0.5 group (1.3 ± 1.0 pc/vf, *P* = 0.044). There was no significant difference with RL (0.4 ± 0.3 pc/vf; Table 3, Figure 5). Using Periodic Acid-Schiff stain no tubular atrophy was seen in any of the groups. On Sirius red sections, there was no increase in interstitial fibrous tissue content in all groups.

**Table 3:** Histopathological scoring of osmotic nephrosis-like lesions, proliferating nuclear antigen and activated macrophages

	**HES 200**	**HES 130**	**RL**
OL (score)	2.1 ± 0.6	2.0 ± 0.5	1.1 ± 0.4^a^
PCNA (pc/vf)	18.8 ± 7.1	7.2 ± 1.7^b^	14.1 ± 4.1
Interstitial	18.0 ± 6.9	6.5 ± 1.6^c^	13.5 ± 4.0
Glomerular	1.2 ± 0.7	1.0 ± 0.3	0.9 ± 0.2
ED-1 (pc/vf)	1.3 ± 1.0	0.2 ± 0.04^d^	0.4 ± 0.3

Data are presented as mean ± standard deviation.

^a ^RL *vs*. 10% HES 200/0.5 and RL *vs*. 6% HES 130/0.42; *P* = 0.002

^b ^6% HES 130/0.42 *vs*. 10% HES 200/0.5; *P* = 0.008

^c ^6% HES 130/0.42 *vs*. 10% HES 200/0.5; *P* = 0.006

^d ^6% HES 130/0.42 *vs*. 10% HES 200/0.5; *P* = 0.044

ED-1 = activated macrophages; HES = hydroxyethyl starch; OL = osmotic nephrosis-like lesions; PCNA = proliferating nuclear antigen; RL = Ringer's lactate.

## Discussion

In our study, directly after haemodilution both HES solutions induced a significant decrease in diuresis, Cl_crea _and sodium transport compared with RL. We identified interstitial macrophage infiltration indicating interstitial inflammation and tubular damage as structural alterations which underlie the HES-induced adverse effects on renal function using an isolated porcine renal perfusion model. Furthermore, we demonstrated that 10% HES 200/0.5 caused more interstitial macrophage influx and tubular damage than 6% HES 130/0.42.

The issue of HES-induced effects on renal function continues to be debated. Various investigations found vacuoles in various human tissue specimens [14,15]. These HES-containing vacuoles were called osmotic-like lesions. The OL were suggested to explain the adverse renal effects of HES, although a pathophysiological relationship between the vacuoles and renal dysfunction was not shown. In the situation of kidney transplantation, histological OL of the tubules have been noticed retrospectively in kidney transplant recipients when HES was used for fluid resuscitation of donors with dead brainstems [11]. However, these lesions had no significant effect on the occurrence of delayed graft function and serum creatinine at three and six months post-transplantation. A prospective trial using 6% HES 200/0.62 demonstrated a detrimental effect on initial graft function [12]. Accordingly, in our study the haemodilution with either HES solution directly before kidney retrieval induced a significant reduction of diuresis, Cl_crea _and sodium transport compared with RL. In another retrospective study using 6% HES 200/0.5 did not impair early graft function after kidney transplantation. However, in the retrospective study less HES was administered [16].

There is only limited data available regarding VRS in an isolated renal perfusion model. The model of isolated porcine renal perfusion has been used extensively for the investigation of renal effects of therapeutic interventions [17]. In the study by Hauet and colleagues, a non-specified HES solution was compared with NaCl, gelatine and albumin in an isolated, renal whole blood model over two hours. The authors concluded from their results that HES induces an osmotic nephrosis [18]. Recently, it was shown that large amounts of 6% HES 200/0.5 aggravate macrophage's enzyme-release in patients with impaired renal function possibly resulting in an acquired lysosomal storage disease [19].

Interestingly, in our renal perfusion model vacuolisation of tubular epithelial cell were seen in all groups. They were significantly more present with the application of 10% HES 200/0.5 and 6% HES 130/0.42 compared with RL. However, there were also OL in the RL group, thus the question remains whether these tubular epithelial vacuoles represent renal injury or might be an epiphenomenon without clinical importance.

The increase of beta-NAG levels in all groups indicated that tubular injury had occurred. Using RL in our study, diuresis and Cl_crea _were significantly higher and beta-NAG levels significantly lower comparing with both 10% HES 200/0.5 and 6% HES 130/0.42. Of note, some differences between 6% HES 130/0.42 and RL had gone after four hours. On histological investigations we could identify interstitial proliferation, macrophage influx and tubular damage as structural alterations of the kidney associated with 10% HES 200/0.5-induced adverse renal effects. In addition, significant differences between the two HES solutions with respect to macrophage infiltration could be identified: interstitial inflammation was more pronounced in 10% HES 200/0.5 than in 6% HES 130/0.42. Furthermore, interstitial cell proliferation was significantly higher in 10% HES 200/0.5 than in 6% HES 130/0.42 indicating more pronounced cell activation by 10% HES 200/0.5. The underlying link between macrophages and HES-induced renal failure needs to be elucidated further.

Lang and colleagues demonstrated that a high molecular weight HES preparation (6% HES 670 kD) has no intrinsic non-thiol-dependent anti-inflammatory properties *in vitro *indicating that HES preparations may have pro-inflammatory effects. This phenomenon was not observed with human serum albumin. The binding of neutrophil-derived myeloperoxidase to bovine aortic endothelial cells, a mediator of multiple oxidative and nitric oxide-consuming reactions, was also enhanced [20]. Increased nitric oxide production through inducible nitric oxide synthase activity was revealed to induce decreased expression of tight junction proteins and decreased tight junction localisation in endotoxaemic mice [21]. This was associated with gut epithelial barrier dysfunction evidenced by increased ileal mucosal permeability. On the other hand, the release of endotoxin in sepsis activates leucocyte-endothelial cell adhesion, capillary leakage and changes in vascular micro-haemodynamics [22]. Hoffmann and colleagues showed a reduction of endotoxin-induced leucocyte-endothelial cell interaction in endotoxaemic hamsters using HES 130/0.4, thereby ameliorating endothelial damage [23]. Using a porcine septic shock model, Marx and colleagues demonstrated attenuation of systemic capillary leakage by HES 130/0.42 in comparison with HES 200/0.5 [24]. Hence, there is experimental evidence that different HES solutions may exert different effects on the inflammatory process.

Major limitations of our study were that we compared an iso-oncotic 6% HES 130/0.42 solution (*in vitro *COP 37.8 mmHg) with a hyperoncotic 10% HES 200/0.5 solution (*in vitro *COP 80 to 85 mmHg), thus we cannot differentiate whether the oncotic force, the molecular weight, the degree of molar substitution, molecular size or the combination of all or some factors were determining factors of HES-induced adverse effects on renal function and structure. Further, the model used lacks the volume of distribution and the metabolism of a living organism. Other limitations were the small number of animals per group and the short experimental duration of *ex-vivo *perfusion using a centrifugal blood pump providing non-pulsatile flow. Hence it offers no insights of potential long term-effects of the investigated VRS.

## Conclusions

In conclusion, after haemodilution both HES solutions lead to a significant decrease in diuresis, Cl_clea _and sodium transport compared with RL. We identified renal interstitial proliferation, macrophage infiltration and tubular damage as potential pathological mechanisms of HES-induced adverse effects on renal function using an isolated porcine renal perfusion model. Furthermore, we demonstrated that 10% HES 200/0.5 had more of a pro-inflammatory effect compared with 6% HES 130/0.42 and caused more pronounced tubular damage than 6% HES 130/0.42 and RL.

## Key messages

• Directly after haemodilution both HES solutions lead to a significant decrease in diuresis, Cl_clea _and sodium transport compared with RL.

• 10% HES 200/0.5 caused more interstitial proliferation and macrophage infiltration than 6% HES 130/0.42 in that model.

• In an isolated renal perfusion model, 10% HES 200/0.5 caused more tubular damage compared with 6% HES 130/0.42 and RL.

## Abbreviations

ANOVA: analysis of variance; Beta-NAG: N-acetyl-beta-aminoglucosidase; Cl_Crea_: creatinine clearance; COP: colloid osmotic pressure; ECG: echocardiography; ED-1: marker for macrophage infiltration; H&E: haematoxylin and eosin; HES: hydroxyethyl starch; IOPS: isolated organ perfusion system; NaCl: sodium chloride; OL: osmotic nephrosis-like lesions; PaCO_2_: partial pressure of arterial carbon dioxide; PaO_2_: partial pressure of arterial oxygen; PCNA: proliferating nuclear antigen; P_Crea_: serum creatinine concentration; pc/vf: positive cells/visual field; RL: Ringer's lactate; U_Crea_: urine creatinine concentration; U_Vol_: urine volume during the collection period; VRS: volume replacement solution.

## Competing interests

LH has received lecture fees from BBraun. T-PS has received travel grants from BBraun and Serumwerke Bernburg. KR reports receiving lecture and consulting fees and has received restricted research grants from BBraun. GM has received honoraria for consulting or lecturing, restricted research grants from the following companies: BBraun, Edwards Life Sciences, CSL Behring, Serumwerke Bernburg, Pulsion Medical Systems, Hutchinson Technology, Baxter and Wyeth. All the other authors declare that they have no competing interests.

## Authors' contributions

LH participated in the design of the study, did the investigation and wrote the manuscript. T-PS did the animal preparation and participated in study design. LW constructed the isolated organ perfusion system and managed it. TS participated in the design of the study and performed the statistical analysis. GW participated in the design of the study and helped to draft the manuscript. KA performed the histological part of the investigation. KR and GM had the original idea, designed the study and wrote the manuscript. All authors read and approved the final manuscript.

## References

[B1] Imm A, Carlson RW (1993). Fluid resuscitation in circulatory shock. Crit Care Clin.

[B2] Rivers E, Nguyen B, Ha vstad S, Ressler J, Muzzin A, Knoblich B, Peterson E, Tomlanovich M, Early goal-directed therapy collaborative group (2001). Early goal-directed therapy in the treatment of severe sepsis and septic shock. N Engl J Med.

[B3] Dellinger RP, Levy MM, Carlet JM, Bion J, Parker MM, Jaeschke R, Reinhart K, Angus DC, Brun-Buisson C, Beale R, Calandra T, Dhainaut JF, Gerlach H, Harvey M, Marini JJ, Marshall J, Ranieri M, Ramsay G, Sevransky J, Thompson BT, Townsend S, Vender JS, Zimmerman JL, Vincent JL (2008). Surviving Sepsis Campaign: international guidelines for management of severe sepsis and septic shock: 2008. Intensive Care Med.

[B4] Marx G (2003). Fluid therapy in sepsis with capillary leakage. Eur J Anaesthesiol.

[B5] Schortgen F, Lacherade JC, Bruneel F, Cattaneo I, Hemery F, Lemaire F, Brochard L (2001). Effects of hydroxyethylstarch and gelatin on renal function in severe sepsis: a multicentre randomised study. Lancet.

[B6] Boldt J (2001). Hydroxyethylstarch as a risk factor for acute renal failure in severe sepsis. Lancet.

[B7] Godet G (2001). Hydroxyethylstarch as a risk factor for acute renal failure in severe sepsis. Lancet.

[B8] Gosling P, Rittoo D, Manji M, Mahmood A, Vohra R (2001). Hydroxyethylstarch as a risk factor for acute renal failure in severe sepsis. Lancet.

[B9] Brunkhorst FM, Engel C, Bloos F, Meier-Hellmann A, Ragaller M, Weiler N, Moerer O, Gruendling M, Oppert M, Grond S, Olthoff D, Jaschinski U, John S, Rossaint R, Welte T, Schaefer M, Kern P, Kuhnt E, Kientopf M, Hartog C, Natanson C, Loeffler M, Reinhart K, for the German Competence Network Sepsis (SepNet) (2008). Intensive insulin therapy and pentastarch resuscitation in severe sepsis. N Engl J Med.

[B10] Sakr Y, Payen D, Reinhart K, Sipmann FS, Zavala E, Bewley J, Marx G, Vincent JL (2007). Effects of hydroxyethyl starch administration on renal function in critically ill patients. Br J Anaesth.

[B11] Legendre C, Thervet E, Page B, Percheron A, Noel LH, Kreis H (1993). Hydroxyethylstarch and osmotic-nephrosislike lesions in kidney transplantation. Lancet.

[B12] Cittanova ML, Leblanc I, Legendre C, Mouquet C, Riou B, Coriat P (1996). Effect of hydroxyethylstarch in brain-dead kidney donors on renal function in kidney-transplant recipients. Lancet.

[B13] Pearson ES, Please NW (1975). Relation between the shape of population distribution and the robustness of four simple test statistics. Biometrika.

[B14] Sirtl C, Laubenthal H, Zumtobel V, Kraft D, Jurecka W (1999). Tissue deposits of hydroxyethyl starch (HES): dose-dependent and time-related. Br J Anaesth.

[B15] Auwerda JJ, Wilson JH, Sonneveld P (2002). Foamy macrophage syndrome due to hydroxyethyl starch replacement: a severe side effect in plasmapheresis. Ann Intern Med.

[B16] Deman A, Peeters P, Sennesael J (1999). Hydroxyethyl starch does not impair immediate renal function in kidney transplant recepients: a retrospective, multicentre analysis. Nephrol Dial Transplant.

[B17] Grosse-Siestrup C, Unger V, Fehrenberg C, Baeyer H, Fischer A, Schaper F, Groneberg DA (2002). A model of isolated autologously hemoperfused porcine slaughterhouse kidneys. Nephron.

[B18] Hauet T, Faure JP, Baumert H, Bardou A, Gibelin H, Beguinot S, Germonville T, Hebrard W, Choulet P, Carretier M, Eugene M (1998). Influence of different colloids on hemodynamic and renal functions: comparative study in an isolated perfused pig kidney model. Transplant Proc.

[B19] Auwerda JJ, Leebeek FW, Wilson JH, van Diggelen OP, Lam KH, Sonneveld P (2006). Acquired lysosomal storage caused by frequent plasmapheresis procedures with hydroxyethyl starch. Transfusion.

[B20] Lang JD, Figueroa M, Chumley P, Aslan M, Hurt J, Tarpey MM, Alvarez B, Radi R, Freeman BA (2004). Albumin and hydroxyethyl starch modulate oxidative inflammatory injury to vascular endothelium. Anesthesiology.

[B21] Han X, Fink MP, Yang R, Delude RL (2004). Increased iNOS activity is essential for intestinal epithelial tight junction dysfunction in endotoxemic mice. Shock.

[B22] Schmidt W, Schmidt H, Bauer H, Gebhard MM, Martin E (1997). Influence of lidocaine on endotoxin-induced leukocyte-endothelial cell adhesion and macromolecular leakage in vivo. Anesthesiology.

[B23] Hoffmann JN, Vollmar B, Laschke MW, Inthorn D, Schildberg FW, Menger MD (2002). Hydroxyethyl starch (130 kD), but not crystalloid volume support, improves microcirculation during normotensive endotoxemia. Anesthesiology.

[B24] Marx G, Pedder S, Smith L, Swaraj S, Grime S, Stockdale H, Leuwer M (2006). Attenuation of capillary leakage by hydroxyethyl starch (130/0.42) in a porcine model of septic shock. Crit Care Med.

